# Upadacitinib for the treatment of psoriasiform and spongiotic dermatitis: A multicenter case series

**DOI:** 10.1016/j.jdcr.2024.05.009

**Published:** 2024-05-16

**Authors:** Neda Shahriari, Bruce Strober, Mona Shahriari

**Affiliations:** a Department of Dermatology, Brigham and Women’s Hospital, Harvard Medical School, Boston, Massachusetts; b Department of Dermatology, Yale University, New Haven, Connecticut; c Central Connecticut Dermatology, Cromwell, Connecticut

**Keywords:** dermatitis, eczema, JAK-STAT pathway, psoriasis, psoriasiform, spongiotic, upadacitinib

Psoriasiform and spongiotic dermatitis is a histologic diagnosis characterized by epidermal hyperplasia or regular acanthosis and intraepidermal intercellular edema. Given the overlapping features of psoriasis and eczema, this histologic diagnosis causes a diagnostic and therapeutic dilemma for the clinician. The pathogenesis of psoriasis and eczema is distinct—driven by T helper 17 (Th17) and Th1 T cells in the former and mediated by Th2 cells that produce interleukin 4 (IL-4), IL-5, and IL-13 in the latter. Currently, no clinically available test can assess the cytokine milieu of psoriasiform and spongiotic dermatitis biopsy specimens to better stratify patients as having eczema versus psoriasis. Furthermore, the reliance on clinical presentation can be difficult. Therefore, the clinician often must trial medications targeting either Th2 or Th17 inflammation until a clinical response is achieved. In this vein, there is a critical need for an effective therapy in patients with psoriasiform and spongiotic dermatitis that potentially eliminates therapeutic trial and error.

Upadacitinib inhibits the Janus kinase-signal transducer and activator of transcription pathway and is Food and Drug Administration-approved for the treatment of both atopic dermatitis and psoriatic arthritis. To date, 1 case series details 4 patients achieving remission while receiving upadacitinib for concomitant psoriasis and atopic dermatitis.^1^ However, no report has specifically evaluated the use of upadacitinib in patients presenting with a dermatitis displaying biopsy findings of psoriasiform and spongiotic dermatitis. To this end, we evaluated patients at 2 clinical/academic centers who had biopsy-proven disease. All patients were referred from an outside dermatologist for presumed recalcitrant psoriasis.

Seven patients (6 female; median age, 63 [16-69] years; Table I) with biopsy-proven psoriasiform and spongiotic dermatitis were treated with upadacitinib for at least 16 weeks, between October 2022 and January 2024. All patients had failed topical and/or systemic therapies, displayed pruritus at baseline, and had a Physician’s Global Assessment between 3 and 4 and body surface area ranging from 8% to 33%. After 16 weeks of treatment with upadacitinib (15-30 mg), all patients showed improvement with the Physician’s Global Assessment decreasing to 0 to 2 and body surface area 0% to 15%; representative clinical images are highlighted in Figs 1 and 2. Complete or near resolution of pruritus was noted in all patients.

**Table I:** Clinical characteristics and response to upadacitinib of biopsy-proven cases of psoriasiform and spongiotic dermatitis

Age (y)	Gender	FST	Initial clinical suspicion	Prior treatments∗	Presence of eosinophils on pathology?	Baseline PGA	Baseline BSA (%)	Posttreatment PGA	Posttreatment BSA (%)	UPA dose (mg)
16	F	II	Plaque psoriasis	Ustekinumab, ixekizumab, certolizumab, guselkumab, risankizumab, deucravacitinib	No	4	10	0	0	30
68	F	II	Plaque psoriasis	TCS, ixekizumab, risankizumab, etanercept	No	4	33	2	15	15
69	F	II	Plaque psoriasis	TCS, roflumilast, tacrolimus	No	3	10	1	5	15
28	F	II	Psoriasis	TCS, guselkumab, apremilast, deucravacitinib	No	3	10	2	5	30
67	M	V	Plaque psoriasis	TCS, antihistamines, doxepin, doxycycline	Rare	4	20	0	0	30
55	F	III	Plaque psoriasis	TCS, MTX, guselkumab, ixekizumab, risankizumab, adalimumab	No	4	8	0	0	15
63	F	I	Guttate psoriasis	TCS, guselkumab	Few	4	15	0	0	15

*BSA*, Body surface area; *F*, female; *FST*, Fitzpatrick skin type; *M*, male; *MTX*, methotrexate; *PGA*, Physician’s Global Assessment; *TCS*, topical corticosteroids; *UPA*, upadacitinib.

∗ Prior medications were considered a failure if taken for at least 12 weeks without improvement in disease or not tolerated by the patient for any reason.

**Fig 1:**
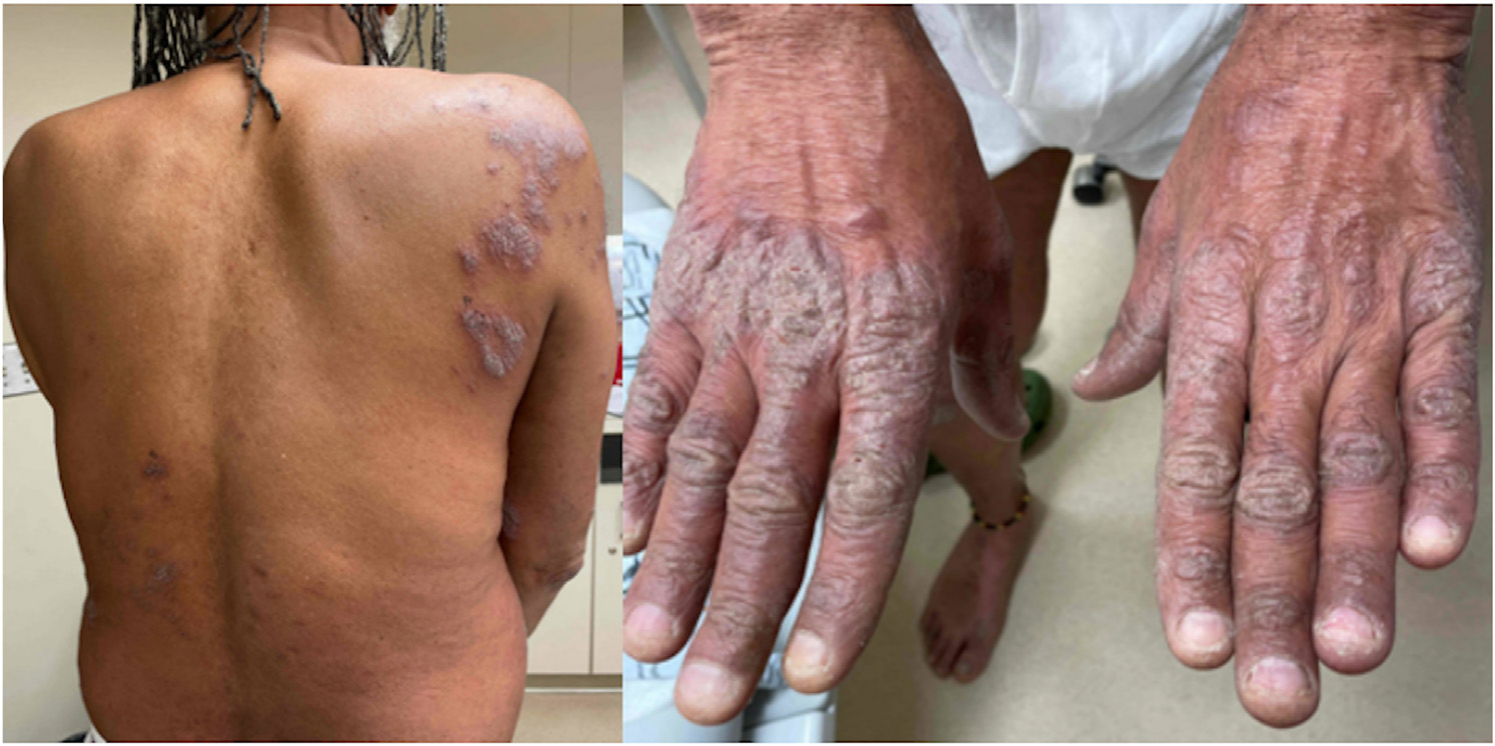
Representative clinical images of 1 patient with biopsy-proven psoriasiform and spongiotic dermatitis on presentation. Note the well-demarcated erythematous plaques with silvery scale involving the back and dorsal aspect of the hands.

**Fig 2:**
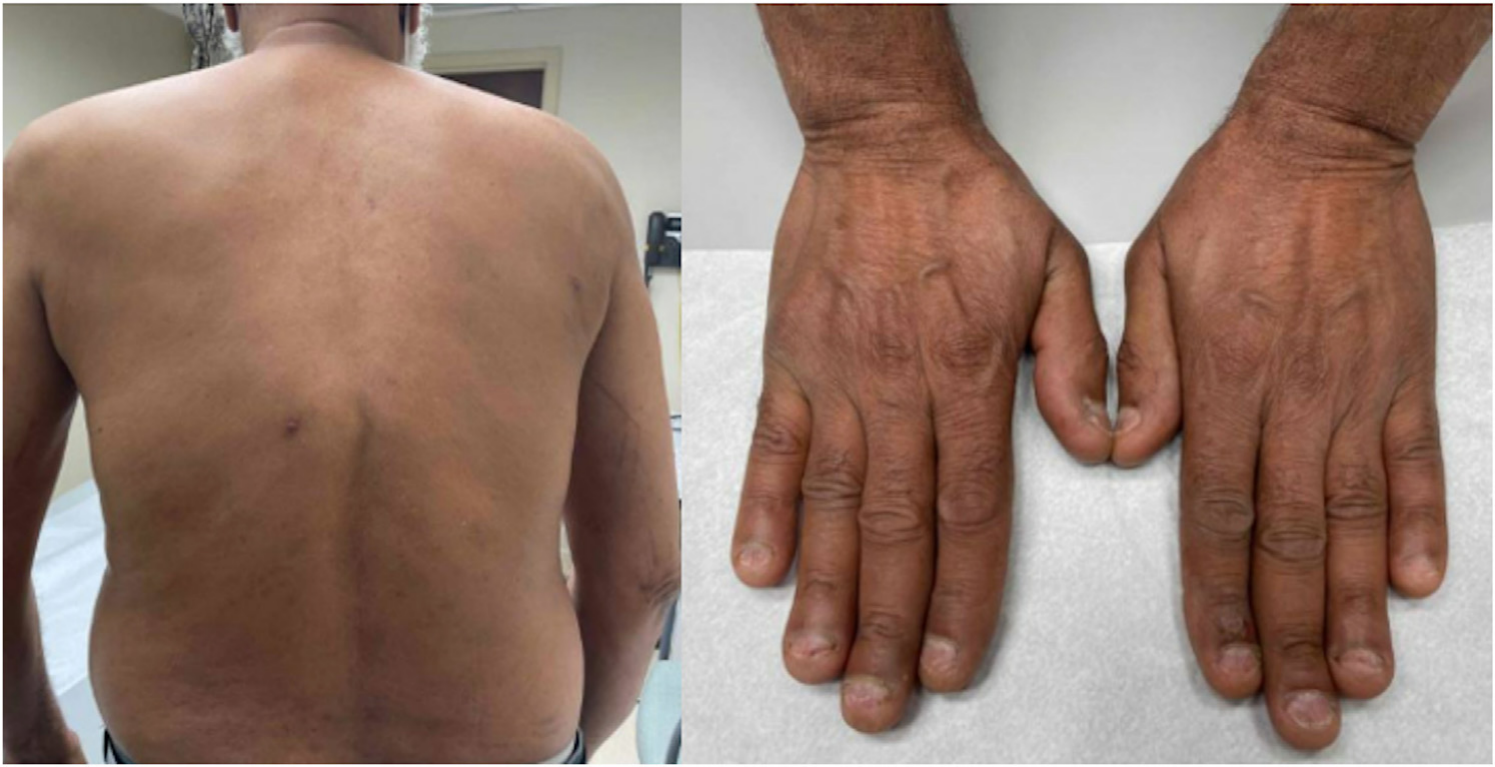
Clinical images 16 weeks after treatment with upadacitinib. Note complete resolution of skin lesions on the back and dorsal aspect of the hands while on treatment.

Psoriasiform and spongiotic dermatitis causes a diagnostic and therapeutic dilemma for the dermatologist who must rely on trialing different medications that target both eczema and psoriasis. Although the underlying pathophysiology of this entity remains ill-defined, a previous study evaluating biopsies from psoriasis and eczema found that the upregulation of both IL-36 and beta-defensin 2 can induce IL-17 pathway activation and is seen preferentially in psoriatic lesions as compared with eczema.^2^ In this same study, application to psoriasiform and spongiotic dermatitis biopsy specimens showed variable expression pattern,^2^ suggesting some cases are more psoriatic than spongiotic. It also has been suggested that patients with this histopathologic diagnosis may have a distinctly hybrid disease, as a subset of Asian atopic dermatitis patients were shown to have activation of both Th17 and Th2 axes in their affected skin.^3^ Our case series of patients with biopsy-proven psoriasiform and spongiotic dermatitis demonstrated positive responses to upadacitinib, achieving either significant improvement or remission. Regardless of the mechanism underlying the psoriasiform and spongiotic dermatitis, inhibition of the Janus kinase-signal transducer and activator of transcription pathway can help in the management of this disease given its role in IL-23/IL-17 inflammatory axis^4^ and Th2 pathway upregulation.^5^ This case series demonstrates that upadacitinib should be strongly considered as an effective therapy for this uniquely challenging dermatitis.

## Conflicts of interest

Dr Strober is a consultant who has received honoraria from AbbVie, Acelyrin, Alamar, Alumis, Almirall, Amgen, Arcutis, Arena, Aristea, Asana, Boehringer Ingelheim, Kangpu Pharmaceuticals, Bristol Myers Squibb, Capital One, Celltrion, CorEvitas, Dermavant, Imagenebio, Janssen, Leo, Eli Lilly, Maruho, Okura, Meiji Seika Pharma, Protagonist, Monte Carlo, Takeda, Novartis, Pfizer, UCB Pharma, Rapt, Regeneron, Sanofi-Genzyme, SG Cowen, and Union Therapeutics; holds stock options in Connect Biopharma and Mindera Health; serves as a speaker for AbbVie, Arcutis, Dermavant, Eli Lilly, Incyte, Janssen, Regeneron, and Sanofi-Genzyme; acts as a scientific codirector (consulting fee) for the CorEvitas Psoriasis Registry; served as an investigator for CorEvitas Psoriasis Registry; and holds the position of Editor-in-Chief (honorarium) for the Journal of Psoriasis and Psoriatic Arthritis. Dr M. Shahriari is a consultant who has received honoraria from AbbVie, Bristol Myers Squibb, Dermavant, Janssen, Leo Pharma, Lilly USA, Novartis, Ortho Dermatologics, Sanofi-Genzyme, Regeneron, and UCB; serves as a speaker for AbbVie, Bristol Myers Squibb, Lilly USA, Janssen, Dermavant, and Leo Pharma; and served as investigator for AbbVie, CorEvitas Psoriasis Registry, Dermira, Cara, Dermavant, Novartis, Union, and Mindera. Dr N. Shahriari has no conflicts of interest to declare.

## References

[1] Gargiulo L., Ibba L., Pavia G. (2023). Upadacitinib for the treatment of concomitant psoriasis and atopic dermatitis: a case series. J Dermatolog Treat.

[2] Cohen J.N., Bowman S., Laszik Z.G., North J.P. (2020). Clinicopathologic overlap of psoriasis, eczema, and psoriasiform dermatoses: a retrospective study of T helper type 2 and 17 subsets, interleukin 36, and β-defensin 2 in spongiotic psoriasiform dermatitis, sebopsoriasis, and tumor necrosis factor α inhibitor-associated dermatitis. J Am Acad Dermatol.

[3] Noda S., Suárez-Fariñas M., Ungar B. (2015). The Asian atopic dermatitis phenotype combines features of atopic dermatitis and psoriasis with increased TH17 polarization. J Allergy Clin Immunol.

[4] Subramaniam S.V., Cooper R.S., Adunyah S.E. (1999). Evidence for the involvement of JAK/STAT pathway in the signaling mechanism of interleukin-17. Biochem Biophys Res Commun.

[5] Brandt E.B., Sivaprasad U. (2011). Th2 cytokines and atopic dermatitis. J Clin Cell Immunol.

